# The influence of minimally invasive esophagectomy versus open esophagectomy on postoperative pulmonary function in esophageal cancer patients: a meta-analysis

**DOI:** 10.1186/s13019-022-01824-8

**Published:** 2022-06-03

**Authors:** Jingwen Su, Shuang Li, Qiyu Sui, Gongchao Wang

**Affiliations:** School of Nursing and Rehabilitation, Shandong University, Jinan, 250012 Shandong China

**Keywords:** Esophageal cancer, Minimally invasive esophagectomy, Open esophagectomy, Pulmonary function, Meta-analysis

## Abstract

**Objective:**

To compare the influence of minimally invasive esophagectomy (MIE) and open esophagectomy (OE) on postoperative pulmonary function in patients with esophageal cancer.

**Methods:**

Studies about the influence of MIE and OE on postoperative pulmonary function in esophageal cancer patients were searched from PubMed, EMBASE, the Cochrane Library, CNKI, Chinese Science and Technology Journal Database, CBM, and Wanfang Data from inception to March 18, 2021. Meta-analysis was performed using the RevMan 5.3.

**Results:**

This analysis included eight studies, enrolling 264 patients who underwent MIE and 257 patients who underwent OE. The meta-analysis results showed that the MIE group had a higher outcome regarding the percent predicted vital capacity (%VC), forced vital capacity (FVC), forced expiratory volume in 1 s (FEV_1_), and maximum voluntary ventilation (MVV) 1 month after surgery than the OE group. In addition, those who underwent MIE had lower ΔVC%, ΔFVC, and ΔFEV_1_ between pre-operation and 1 month after surgery than those who underwent OE. There is no statistical difference between the two groups in ΔMVV.

**Conclusion:**

Compared with OE, MIE has a more protective effect on postoperative pulmonary function. However, due to the small number of included literature and all cohort studies, this finding needs to be validated with larger samples and higher quality RCT studies.

## Introduction

Esophageal cancer is a malignant tumor arising in the epithelium or glands of the esophageal mucosa. According to the Global Cancer Statistics 2018, esophageal cancer ranked 7th in new cancer cases and 6th in deaths [1]. At present, the clinical treatments of esophageal cancer mainly include surgery, radiotherapy, and chemotherapy, in which surgery remains the cornerstone of current therapy. Open esophagectomy, as the main surgical procedure, has the advantages of thorough lymph dissection, but thoracotomy incision has a great influence on pulmonary function. Studies reported a dramatic decrease in pulmonary function of about 30% due to chest wall injury from surgery, mechanical lung injury, and painful irritation in the early postoperative period [2].

In recent years, with the gradual maturation of surgical techniques, MIE has become one of the mainstream ways to treat esophageal cancer. Although MIE reduces the mechanical crush on lung tissue during surgery, there are still effects on postoperative pulmonary function of patients. The most common postoperative complication of esophagectomy and the leading cause of postoperative death is pulmonary complications, and a reduction in complications has a potentially positive impact on survival and timing of tumor recurrence after esophagectomy [3–5]. Since the severity of impaired pulmonary function is significantly associated with the occurrence of pulmonary complications, attention to changes in pulmonary function should be a priority in esophageal cancer patients [3].

Previous researches mostly focused on the effects of different surgical methods on perioperative pulmonary function in esophageal cancer patients. However, in the early postoperative period, the integrity of the chest wall is disrupted by OE, and several studies have confirmed that MIE is more effective in improving pulmonary function in patients with esophageal cancer compared with OE [6, 7]. Nevertheless, some studies have found no statistical difference between the MIE group and the OE group in some pulmonary function indicators at 1 month after operation [8, 9]. Conclusions are still controversial. This meta-analysis was conducted to compare the effects of MIE versus OE on pulmonary function in patients with esophageal cancer at 1 month after surgery and to provide a scientific basis for clinical use.

## Methods

### Literature search strategy

Literature was identified by searching databases including PubMed, EMBASE, the Cochrane Library, CNKI, Chinese Science and Technology Journal Database, CBM, and Wanfang Data up to March 2021. Search terms used in the literature search include “esophageal neoplasm, esophageal carcinoma, esophagus neoplasm, esophagus cancer, oesophagus cancer, oesophageal cancer, esophagectomy, oesophagectomy, minimally invasive esophagectomy, open esophagectomy, robot assist, resection, lung function, pulmonary function, and respiratory function.” This review was registered in the International Prospective Register of Systematic Reviews, PROSPERO (Registration No. CRD42021245622).

### Study selection

Studies included in the meta-analysis had to meet the following characteristics: (1) study type: cohort studies comparing the effects of MIE and OE on post-operative pulmonary function in patients with esophageal cancer; (2) studies written in Chinese or English; (3) study population: patients with esophageal cancer who underwent MIE or OE, no restrictions on the lesion site, pathological type, TNM stage, etc; (4) full article available; and (5) outcomes: the percent predicted vital capacity (%VC), forced vital capacity (FVC), forced expiratory volume in 1 s (FEV_1_), and maximum voluntary ventilation (MVV) in the MIE and OE groups at 1 month after surgery. Two authors eliminated apparently irrelevant literature by skimming the titles and abstracts, and then read the full text further to determine inclusion. In the case of disagreement, it was resolved by discussing the literature among authors.

### Data extraction

All data were extracted independently by two authors to ensure accuracy. If disagreements existed, the investigators resolved them through discussion as well as by seeking third-party opinions. Extracted information included first author name, publication year, age, sex, treatment, TNM stage, number of patients, pathological histological type, and preoperative and 1-month postoperative pulmonary function outcomes.

### Quality assessment

Two investigators used the Newcastle–Ottawa scale (NOS) for assessing the quality of each included cohort study.

### Statistical analysis

Meta-analysis was conducted using the Review Manager (RevMan) 5.3 software. Continuous variables were reported as standardized mean differences (*SMD*) and 95% confidence intervals (CI). The heterogeneity among included studies was assessed using the Chi-square test with a test level of α = 0.1, while the extent of the inconsistency was measured by *I*^2^ statistics. If there was statistical heterogeneity among the studies (a two-sided *P* < 0.1 and *I*^2^ > 50%), the heterogeneity among studies was large. Then the significant clinical heterogeneity was addressed using subgroup analysis or sensitivity analysis, or meta-analysis was performed using a random-effects model. Conversely, there was no statistical heterogeneity among studies (a two-sided *P* ≥ 0.1 and *I*^2^ ≤ 50%), and a fixed-effects model was used for analysis. A two-sided *P* < 0.05 indicates that the difference is statistically significant.

## Results

### Selection of eligible studies

**Fig. 1:**
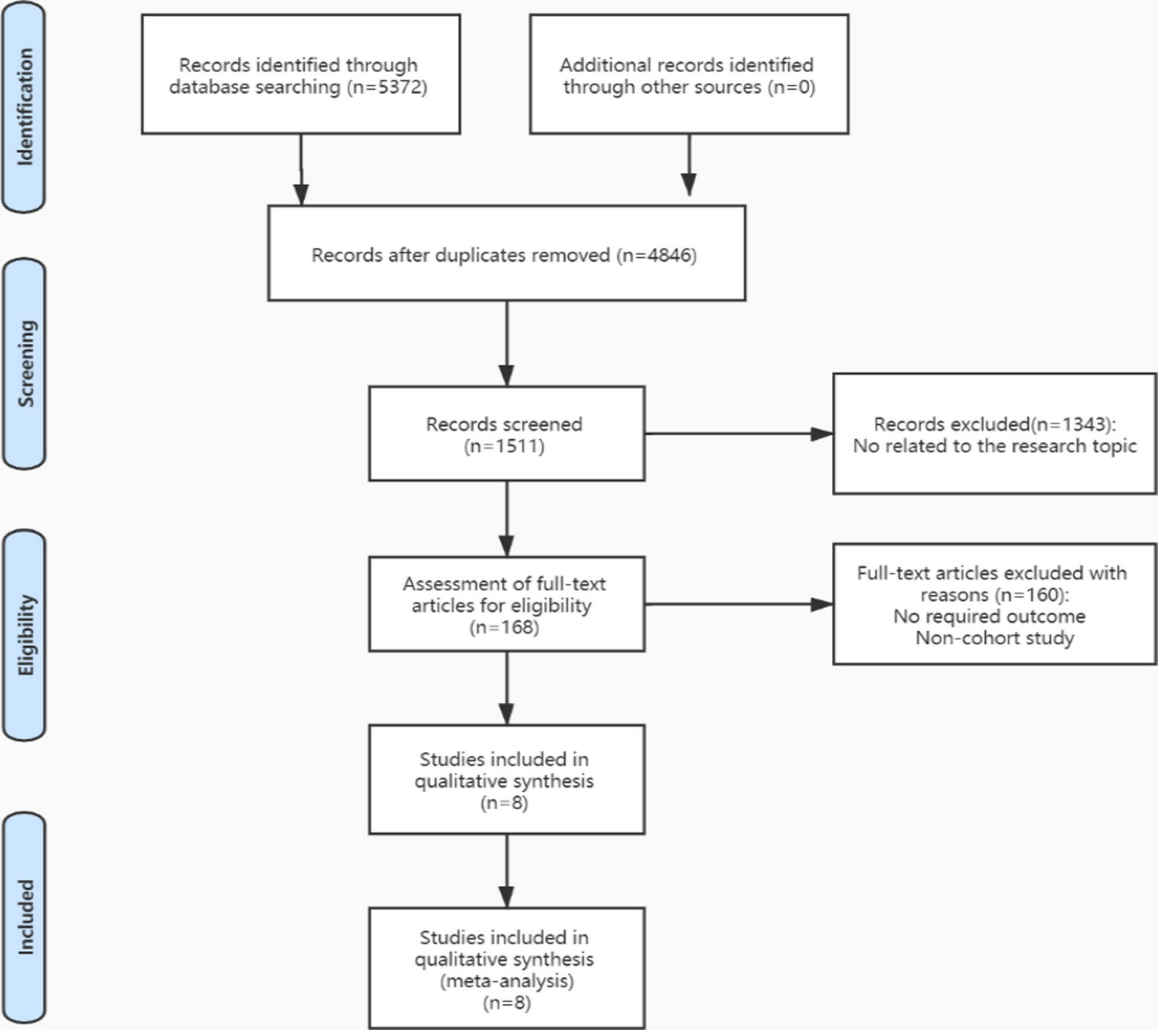
PRISMA flowchart of the literature search strategy

Through the search, 5372 potential studies were identified. 8 studies were eligible for qualitative analysis [6, 7, 10–15] (Fig. 1).

### Basic characteristics and risk of bias

In total, 8 cohort studies involving 521 participants are included in this review. MIE was performed in 264 patients and OE was performed in 257 patients. The basic characteristics and risk of bias results of 8 cohort studies evaluated using the NOS scale are shown in Table 1.

**Table 1:** Basic characteristics and risk of bias

Authors (year)	country	No. of cases		Sex ratio	Average age		Pathological characteristic adenocarcinoma/squamous cell carcinoma/other	TNM staging	Surgical procedure		Postoperative pulmonary function indicators	Preoperative pulmonary function indicators	NOS
		M	O	M/F	M	O							
Li et al. [7]	China	34	35	48/21	NA	NA	0/69/0	I–IIB	MIE	OE	①	NA	7
Wang [6]	China	43	35	46/32	64.89 ± 3.09	65.17 ± 2.95	48/20/10	I–III	MIE	OE	②③	②③	7
Han et al. [10]	China	32	33	45/20	66.8 ± 4.5	66.2 ± 4.3	NA	I–IIB	MIE	OE	②③④	②③④	7
Shi et al. [11]	China	30	30	34/26	61.2 ± 5.3	60.9 ± 5.7	40/18/2	I–III	MIE	OE	②③	②③	7
Gao [12]	China	34	34	48/20	54.92 ± 6.14	55.84 ± 6.32	NA	I–IIB	MIE	OE	②③	②③	7
Wu et al. [13]	China	39	31	34/36	53.52 ± 8.63	52.44 ± 8.33	10/60/0	I–III	MIE	OE	②③	②③	7
Li [14]	China	30	30	37/23	60.13 ± 1.34	60.24 ± 1.17	0/60/0	NA	MIE	OE	①	①	7
Taguchi [15]	Japan	22	29	44/7	61.6 ± 9.3	61.7 ± 6.4	0/51/0	0–III	MIE	OE	①②③④	②③④	6

M: Minimally invasive esophagectomy; O: Open esophagectomy; NA: not available. ① %VC; ② FVC; ③ FEV_1_; ④ MVV

### Comparison of pulmonary function at 1 month after surgery between the MIE and the OE

#### %VC

**Fig. 2:**
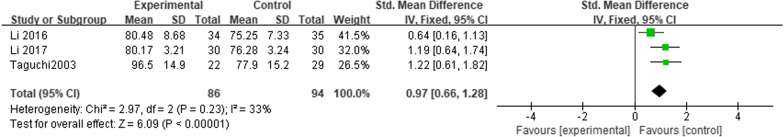
Meta-analysis comparing the effects of MIE and OE on %VC at 1 month after esophagectomy

Three studies reported %VC at 1 month after esophagectomy [7, 14, 15]. This analysis included 180 patients. The heterogeneity test showed *I*^2^ = 33% (*P* = 0.23), and a fixed-effects model was applied. The results suggested that the patients who underwent MIE had higher %VC readings at 1 month after surgery compared with those who underwent OE (*SMD* = 0.97; 95% CI 0.66, 1.28; *P* < 0.00001) (Fig. 2).

#### FVC

**Fig. 3:**
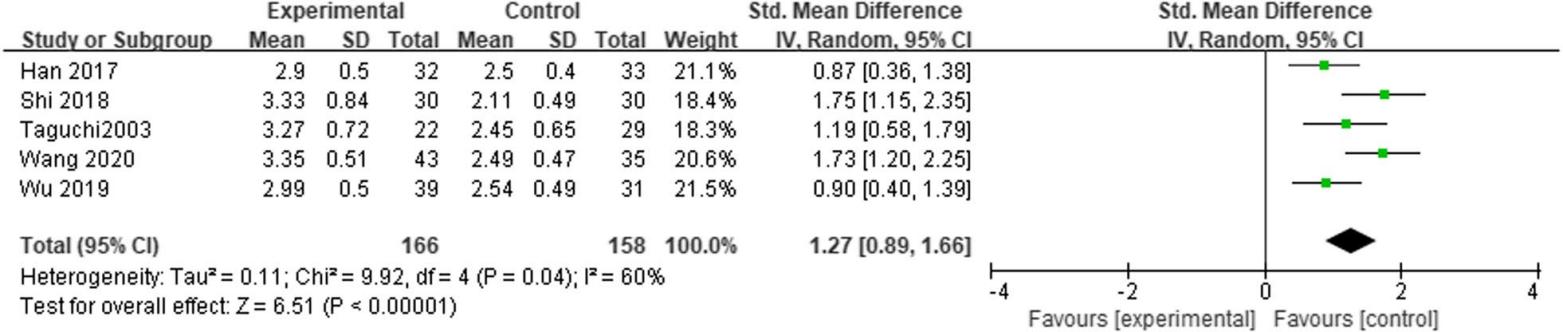
Meta-analysis comparing the effects of MIE and OE on FVC at 1 month after esophagectomy

Six studies reported FVC at 1 month after esophagectomy [6, 10–13, 15]. This analysis included 392 patients. The heterogeneity test showed *I*^2^ = 87% (*P* < 0.00001), and a random-effects model was considered. The results suggested that the patients who underwent MIE had higher FVC readings at 1 month after surgery compared with those who underwent OE (*SMD* = 1.60; 95% CI 0.96, 2.24; *P* < 0.00001). Sensitivity analysis was conducted by the omission of every single study to evaluate the stability of results. Sensitivity analysis suggested that heterogeneity was significantly reduced after excluding one study [12] with a significantly positive outcome, and further meta-analysis using a random-effects model still showed that the patients who underwent MIE had higher FVC readings at 1 month after surgery compared with those who underwent OE. (SMD = 1.27; 95% CI 0.89, 1.66; *P* < 0.00001) (Fig. 3).

#### FEV_1_

**Fig. 4:**
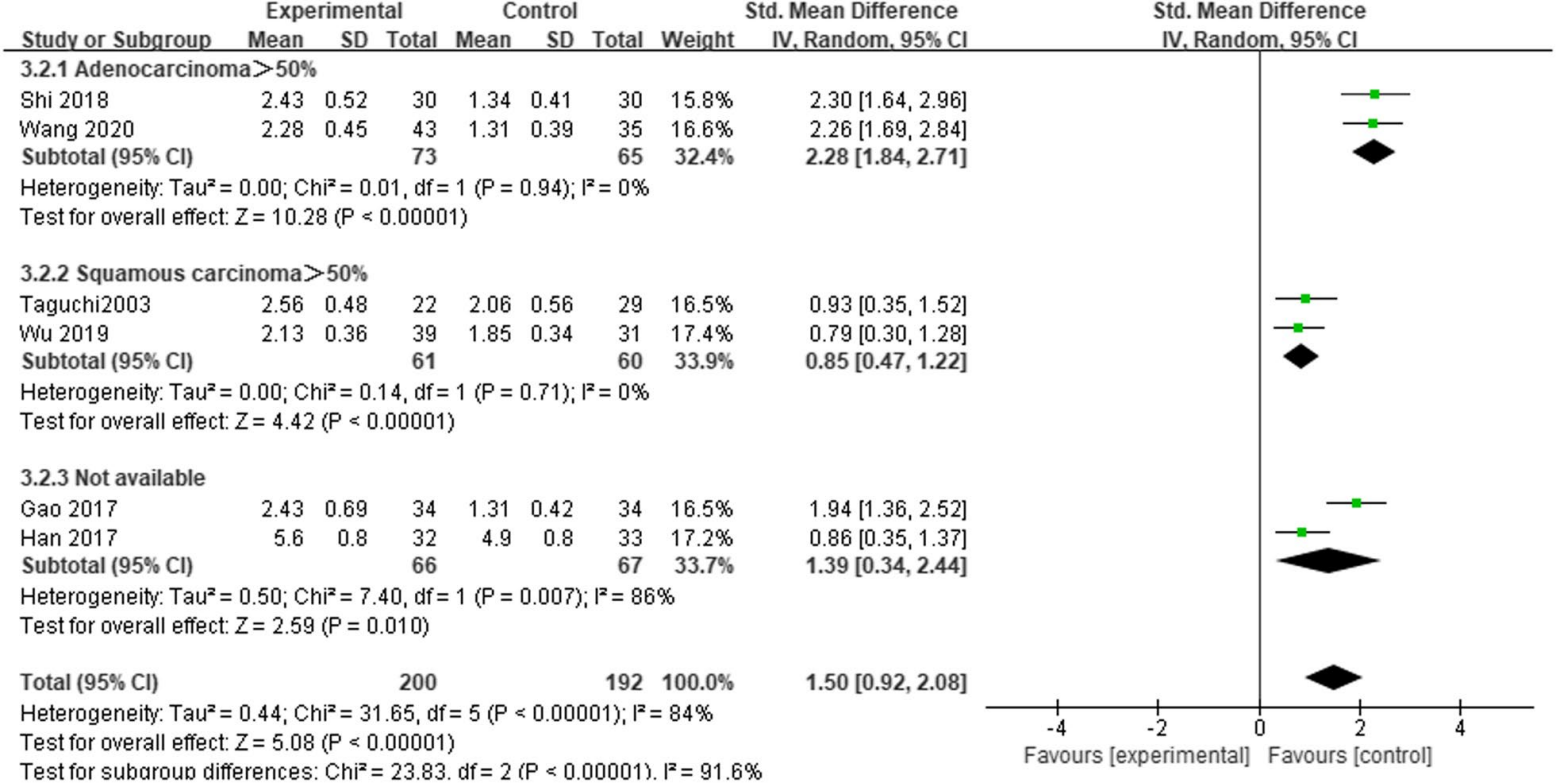
Meta-analysis comparing the effects of MIE and OE on FEV_1_ at 1 month after esophagectomy

Six studies reported FEV_1_ at 1 month after esophagectomy [6, 10–13, 15]. This analysis included 392 patients. Heterogeneity was large among studies (*P* < 0.00001, *I*^2^ = 84%). Subgroup analysis according to the percentage of pathological histological types showed that the patients who underwent MIE had higher FEV_1_ readings at 1 month after surgery compared with those who underwent OE in any subgroup (*SMD* = 2.28; 95% CI 1.84, 2.71; *P* < 0.00001. *SMD* = 0.85; 95% CI 0.47, 1.22; *P* < 0.00001. *SMD* = 1.39; 95% CI 0.34, 2.44; *P* = 0.01) (Fig. 4).

#### MVV

**Fig. 5:**
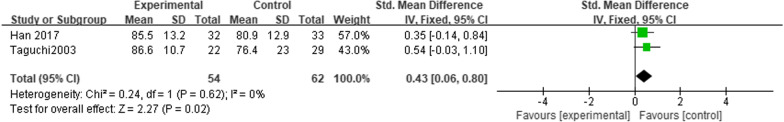
Meta-analysis comparing the effects of MIE and OE on MVV at 1 month after esophagectomy

Two studies reported MVV at 1 month after esophagectomy [10, 15]. This analysis included 116 patients. The heterogeneity test showed *I*^2^ = 0% (*P* = 0.62), and a fixed-effects model was considered. The results suggested that the patients who underwent MIE had higher MVV readings at 1 month after surgery compared with those who underwent OE (*SMD* = 0.43; 95% CI 0.06, 0.80; *P* = 0.02) (Fig. 5).

### Comparison of preoperative and 1-month postoperative pulmonary function index differences between the MIE and the OE

#### Δ%VC

**Fig. 6:**
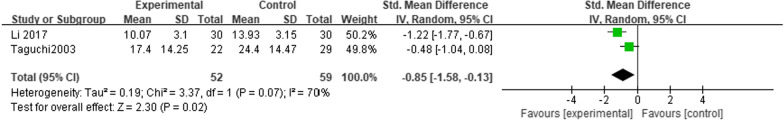
Meta-analysis comparing the effects of MIE and OE on Δ%VC

Two studies reported %VC outcomes before and 1 month after esophagectomy [14, 15]. This analysis included 111 patients. The heterogeneity test showed *I*^2^ = 70% (*P* = 0.07), and a random-effects model was considered. The results suggested that the patients who underwent MIE had lower Δ%VC readings at 1 month after surgery compared with those who underwent OE (*SMD* =  − 0.85; 95% CI − 1.58, − 0.13; *P* = 0.02) (Fig. 6).

#### ΔFVC

**Fig. 7:**
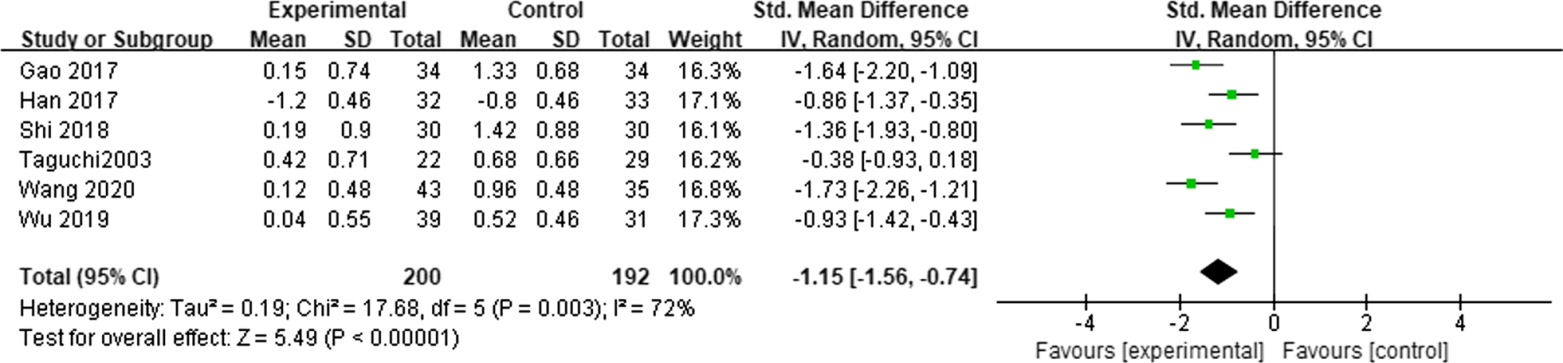
Meta-analysis comparing the effects of MIE and OE on ΔFVC

Six studies reported FVC outcomes before and 1 month after esophagectomy [6, 10–13, 15]. This analysis included 392 patients. The heterogeneity test showed *I*^2^ = 72% (*P* = 0.003), and a random-effects model was considered. The results suggested that the patients who underwent MIE had lower ΔFVC readings at 1 month after surgery compared with those who underwent OE (*SMD* = − 1.15; 95% CI − 1.56, − 0.74; *P* < 0.00001) (Fig. 7).

#### ΔFEV_1_

**Fig. 8:**
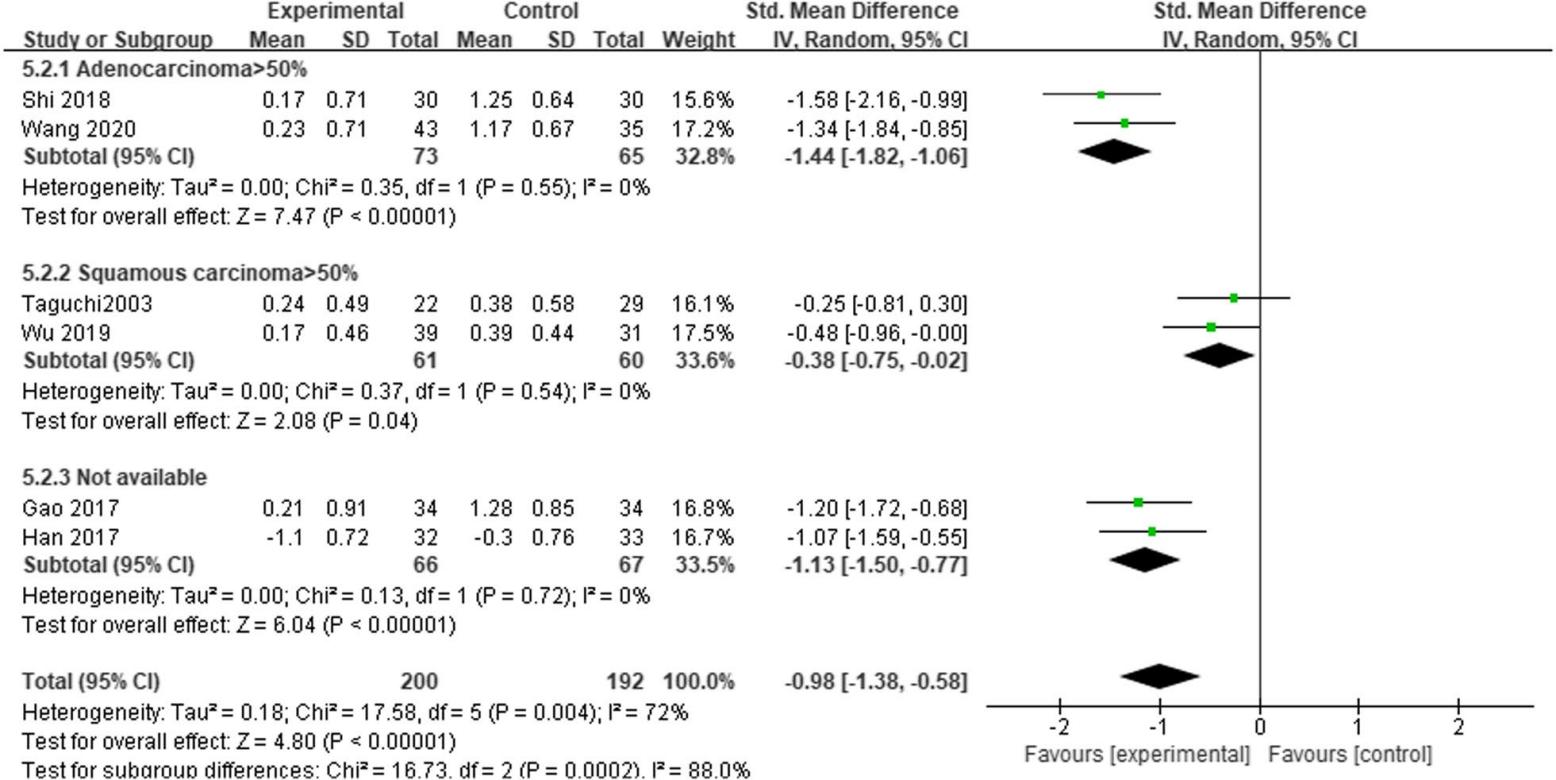
Meta-analysis comparing the effects of MIE and OE on ΔFEV_1_

Six studies reported FEV_1_ outcomes before and 1 month after esophagectomy [6, 10–13, 15]. This analysis included 392 patients. Heterogeneity was large among studies (*P* = 0.004, *I*^2^ = 72%). Subgroup analysis according to the percentage of pathological histological types showed that the patients who underwent MIE had lower ΔFEV_1_ readings at 1 month after surgery compared with those who underwent OE in any subgroup (*SMD* =  − 1.44; 95%CI − 1.82, − 1.06; *P* < 0.00001. *SMD* =  − 0.38; 95% CI − 0.75, − 0.02; *P* = 0.04. *SMD* = − 1.13; 95% CI − 1.50, − 0.77; *P* < 0.00001) (Fig. 8).

#### ΔMVV

**Fig. 9:**
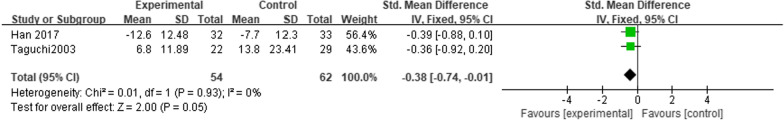
Meta-analysis comparing the effects of MIE and OE on ΔMVV

Two studies reported MVV outcomes before and 1 month after esophagectomy [10, 15]. This analysis included 116 patients. The heterogeneity test showed *I*^2^ = 0% (*P* = 0.93), and a fixed-effects model was considered. The results suggested that the ΔMVV readings at 1 month after surgery were not statistically different between patients who underwent MIE and those who underwent OE (*SMD* =  − 0.38; 95% CI − 0.74, − 0.01; *P* = 0.05). (Fig. 9).

## Discussion

It is well known that esophageal cancer is one of the most common malignancy cancers in the world. Squamous cell carcinoma is the most common histologic type of esophageal cancer, mainly affecting developing countries, while adenocarcinoma is gradually becoming a more common histologic type in developed countries [16]. Esophagectomy is the standard treatment for resectable localized esophageal cancer [17]. Open esophagectomy is more invasive, and in recent years, MIE with the advantages of less trauma and quicker recovery has been favored by more surgeons. However, there is controversy regarding the effect of MIE and OE on postoperative pulmonary function in patients with esophageal cancer [18, 19].

The meta-analysis results showed that the patients who underwent MIE had higher %VC, FVC and FEV_1_ readings at 1 month after surgery and lower ΔVC%, ΔFEV_1_ and ΔFVC between pre-operation and 1 month after surgery compared with those who underwent OE, indicating that MIE was more protective of pulmonary function.

There are several reasons that could explain the results. First, the superiority of pulmonary function in patients after esophagectomy depends on the integrity of the thorax and respiratory muscles, the patency of the airway and the compliance of the thorax with the lung tissue [20]. OE destroys the integrity of the respiratory muscles and the diaphragm, which is the core respiratory muscle that performs 50–80% of the inspiratory function [2]. MIE causes less damage to the chest wall muscles and preserves the integrity and compliance of the thorax while further reducing the impact on surrounding tissue traction and abdominal breathing [21, 22]. Secondly, MIE is less likely to strain the heart and lungs and damage the thoracic duct, while reducing the release of inflammatory factors and stress response, which can shorten the recovery time of pulmonary function of patients [23]. Conversely, the direct compression of the heart and lung hilum during OE causes respiratory dysfunction, decreased lung tissue volume and impaired diaphragm integrity in patients, which impairs pulmonary function [24]. Thirdly, postoperative pain causes the body to release endogenous substances such as catecholamines, aldosterone, cortisol and antidiuretic hormones, and activates the renin-angiotensin system. These hormones act on myocardial and vascular smooth muscle, causing sodium-water retention in the body, resulting in an increase in extravascular lung water (EVLW), which in turn leads to an abnormal ventilation/blood flow ratio. Finally, postoperative incisional pain after OE greatly limits the cough and expectoration of the patients, and increased alveolar exudate causes postoperative obstructive respiratory impairment, which impairs pulmonary ventilation and exchange function [25]. In addition, incisional pain affects forced breathing, resulting in a decrease in exhaled air and a significant increase in residual air.

Meta-analysis results showed that the patients who underwent MIE had higher FEV_1_ readings at 1 month after surgery and lower ΔFEV_1_ between pre-operation and 1 month after surgery compared with those who underwent OE in any subgroup. The Cochrane Handbook mentions that when it comes to comparisons between subgroups, non-overlap of the confidence intervals indicates statistical significance. The results of this study showed no overlap in confidence intervals between the subgroup with a high proportion of adenocarcinoma and the subgroup with a high proportion of squamous carcinoma. Previous studies have found differences in lymph node metastasis between esophageal adenocarcinoma and esophageal squamous cell carcinoma [26]. Early-stage squamous carcinoma is more likely to invade the submucosa than adenocarcinoma, and if it does, squamous carcinoma is more likely to involve the lymph nodes [27]. It has been suggested that more extensive lymphadenectomy not only offers no advantage for survival, but is also more surgically invasive [28]. Some studies included in the meta-analysis did not focus on patients with a specific pathological tissue type, and more convincing clinical trials could be conducted in the future to further compare the effects of different surgical approaches on pulmonary function in patients with different pathological histological types.

MVV correlates with respiratory muscle strength, thoracic elasticity, lung tissue elasticity and airway resistance, and is a comprehensive index for evaluating pulmonary ventilation reserve. The results suggested that the ΔMVV readings were not statistically different between patients who underwent MIE and those who underwent OE. The *p*-value was at the critical value for testing the presence of statistical differences, which may be related to the small number of included studies analyzing MVV readings and requires further expansion of the sample size for determination.

Due to time and effort constraints, only a few studies have focused on the long-term effects of different esophagectomy procedures on pulmonary function in patients with esophageal cancer. Chen [29] and xie [30] have demonstrated that the patients who underwent MIE had higher FEV_1_ readings at 3 months after surgery compared with those who underwent OE. In addition, a retrospective study that included 53 patients who underwent MIE and 34 patients who underwent OE found the decreases of VC and FEV_1_ were significantly lower in the MIE group than in the OE group at 1 year after surgery [31]. This likely implies that the recovery of respiratory muscle function and the improvement of thoracic flexibility and lung tissue elasticity after surgery in patients who underwent different esophagectomy procedures require a long-term process; whereas MIE is more protective for long-term pulmonary function.

## Limitations

First, all articles included in the meta-analysis were cohort studies, which are susceptible to recall and selection bias. Furthermore, the meta-analysis only focused on studies published in Chinese and English, which may have influenced the results. Third, owing to the few studies included in meta-analysis, we did not make funnel plots to assess possible publication bias and missed some indicators such as DLCO% and percentage of the indicators that are more reliable for predicting changes in pulmonary function. Fourth, the analysis results could also be affected by the technique of surgeons, as well as preoperative co-morbidities, and postoperative nutritional support, but this information was not available to us. Finally, different surgical approaches or anastomosis sites may affect the postoperative pulmonary function. For example, the Mckeown procedure is mainly performed through a neck-right thoracic-epigastric incision and anastomosis in the neck, while the modified Ivor-Lewis procedure was performed mainly in the left lateral position with two right thoracic-abdominal incisions. It was found that the modified Ivor-Lewis procedure had little effect on postoperative pulmonary function, because this procedure did not make a cervical incision, thus avoiding damage to tracheal blood flow and protecting pulmonary function [32]. In the future, relevant conclusions need to be further clarified.

## Conclusion

The results showed that the patients who underwent MIE had higher %VC, FVC, FEV_1_ and MVV readings at 1 month after surgery and lower ΔVC%, ΔFEV_1_ and ΔFVC between pre-operation and 1 month after surgery compared with those who underwent OE, indicating MIE was more beneficial for pulmonary function. The effect of different esophagectomies on pulmonary function may also be related to the pathological tissue types. Multicenter RCT trials are needed to continue to further explore MIE and OE on long-term pulmonary function in patients with esophageal cancer.

## Data Availability

The current study was based on the results of relevant published studies.

## Abbreviations

MIE: Minimally invasive esophagectomy
OE: Open esophagectomy
%VC: The percent predicted vital capacity
FVC: Forced vital capacity
FEV_1_: Forced expiratory volume in 1 s
MVV: Maximum voluntary ventilation
SMD: Standardized mean differences
CI: Confidence interval
